# Report of Deaths in the City of Buffalo for the Month of October, 1862

**Published:** 1862-12

**Authors:** Sandford Eastman

**Affiliations:** Health Physician


*Report of Deaths in the City of Buffalo for the month of October*, 1862.

Abscess of liver 1, Accident 1, do by drowning 2, Apoplexy, cerebral 1, Cancer 1, do of the stomach 1, Cholera infantum 4, Consumption 18, Convulsions 9, Croup 5, Debility 1, Delirium tremens 2, Dentition 2, Diarrhœa 10, Disease of the brain 1, do heart 2, do liver 1, do stomach 1, do spine 1 Diphtheria 4, Dropsy abdominal 2, do chest 1, Dysentery 4, Epilepsy 1, Erysipelas 1, Fever puerperal 1, do scarlet 7, do typhoid 4, Gun-shot wound 1, Hæmorrhage 1, Hæmmorrhoids 1, Inflammation of the bowels 1, do brain 2, do and meninges 6 do liver 1, do lungs 3, do peritoneum 1, Insanity 3, Marasmus 3, Measles 2, Old age 10, Paralysis 1, Pyaemia 1, Scrofula 2, Syphilis 1, Unknown 5. Deaths from disease 134. Still-born 4.

SANDFORD EASTMAN, M. D., Health Physician.

